# Does Diabetes Mellitus Alter the Onset and Clinical Course of Vascular Dementia?

**DOI:** 10.3233/BEN-2010-0281

**Published:** 2010-11-23

**Authors:** Santosh B. Murthy, Ali Jawaid, Salah U. Qureshi, Yogeshwar Kalkonde, Andrew M. Wilson, Michael L. Johnson, Mark E. Kunik, Paul E. Schulz

**Affiliations:** ^1^Department of NeurologyBaylor College of MedicineHoustonTXUSA; ^2^Neurology Care LineThe Michael E. DeBakey VA Medical CenterHoustonTXUSA; ^3^Menninger Department of Psychiatry and Behavioral SciencesBaylor College of MedicineHoustonTXUSA; ^4^Center for Quality of Care and Utilization StudiesVeterans Affairs Medical CenterHoustonTXUSA; ^5^University of HoustonCollege of PharmacyHoustonTXUSA; ^6^VA South Central Mental Illness Research Education and Clinical CenterHoustonTXUSA

## Abstract

*Background:* Vascular dementia (VaD) is the second most common dementing illness. Multiple risk factors are associated with VaD, but the individual contribution of each to disease onset and progression is unclear. We examined the relationship between diabetes mellitus type 2 (DM) and the clinical variables of VaD.

*Methods:* Data from 593 patients evaluated between June, 2003 and June, 2008 for cognitive impairment were prospectively entered into a database. We retrospectively reviewed the charts of 63 patients who fit the NINDSAIREN criteria for VaD. The patients were divided into those with DM (VaDDM, n = 29) and those without DM(VaD, *n* = 34). The groups were compared with regard to multiple variables.

*Results:* Patients with DM had a significantly earlier onset of VaD (71.9 ± 6.54 vs. 77.2 ± 6.03,*p* < 0.001), a faster rate of decline per year on the mini mental state examination (MMSE; 3.60 ± 1.82 vs. 2.54 ± 1.60 points, *p* = 0.02), and a greater prevalence of neuropsychiatric symptoms at the time of diagnosis (62% vs. 21%, *p* = 0.02).

*Conclusions:* A history of premorbid DM was associated with an earlier onset and faster cognitive deterioration in VaD. Moreover, DM was associated with neuropsychiatric symptoms in patients with VaD. A larger study is needed to verify these associations. It will be important to investigate whether better glycemic control will mitigate the potential effects of DM on VaD.

## 1. Introduction

Vascular dementia is the second most common type of dementia in the elderly, accounting for 15–20% of all cases [1]. A number of factors are known to increase the risk of vascular dementia, including a history of hypertension, DM, hyperlipidemia, smoking and cardiac conditions [2–4]. Several studies have addressed the contribution of each risk factor to VaD and the question of which, if any, predict the rate of cognitive decline in VaD.

It has been difficult to establish the contribution of each vascular risk factor to VaD because they often occur concurrently in the elderly. DM is one risk factor for VaD and Alzheimer's disease (AD) [5–7]. Kloppenborg et al. reviewed the incidence of both AD and VaD in relation to DM, hypertension, dyslipidemia and obesity, and concluded that DM conveys the highest risk of both dementias in the elderly, whereas hypertension has the greatest contribution in middle-aged individuals [8]. In addition to conferring a greater risk to both of these dementias, DM is also implicated in worsening the course of AD where it is associated with an earlier onset of cognitive symptoms and more rapid cognitive deterioration [9]. It has also been noted that the presence of co-morbid DM can result in a subtle alteration in the pattern of cognitive dysfunction in AD [10].

[1]These authors contributed equally to this work.
[2]Present affiliation: Department of Neurology, UT Health, Houston, TX, USA.
*Corresponding author: Paul E. Schulz, M.D., Associate Professor of Neurology, UT Health, 6410 Fannin Suite 1014, Houston, TX 77030, USA. Tel.: +1 832 325 7080; Fax: +1 713 512 2239; E-mail: Paul.E.Schulz@uth.tmc.edu.

ISSN 0953-4180/10/$27.50 © 2010 – IOS Press and the authors. All rights reserved

It has been difficult to establish specific factors that predict cognitive decline in VaD. Chui et al., for example, reviewed 13 studies of the natural history of VaD and concluded that no single factor predicts significantly faster cognitive decline [11]. In another study, age over 80 years was observed to be a significant predictor of faster cognitive decline in patients with VaD and it was suggested that it probably reflected a superimposed neurodegenerative condition [12]. An MRI-based analysis showed that in older individuals, cerebral small-vessel disease enhances cognitive decline, however the association is independent of the influence of selective vascular risk factors [13].

While it has been difficult to identify a specific role for DM in altering the course of VaD, DM has been associated with a worse cognitive profile for another neurodegenerative disorder, amyotrophic lateral sclerosis (ALS). We found that pre-morbid DM was associated with a 4 year later age of onset for the motor findings of ALS, but a worse cognitive profile. (Jawaid et al. *Under Review*)

Considering the associations between DM and dementia, we hypothesized that DM could alter the course and cognitive or behavioral profile for VaD. In order to gain insight into the effects of DM on VaD, we examined VaD in patients with and without DM. Greater insight into the contribution of individual risk factors, like DM, to VaD may have important preventive and prognostic implications.

## 2. Methods

### *2.1. Study design and participants*

Five hundred and ninety-three consecutive patients were evaluated for cognitive impairment between June 2003 and June 2008 in the Cognitive Disorders Clinic at The Michael E DeBakey Veteran Affairs Medical Center (MEDVAMC), Houston, TX, USA.

The patients were labeled with a clinical diagnosis of 'dementia' based on the definition in Diagnostic and Statistical Manual for Mental Disorders IV (DSMIV). Patients were excluded if they gave a positive history of a major psychiatric disorder (unrelated to VaD) or substance abuse. Patients who screened positive for metabolic delirium on routine clinical investigations were also excluded. The remaining patients ($n = 471$) were entered prospectively into a database that, for the purposes of this study, was searched for patients with VaD. Patients were labeled with VaD if they fulfilled the NINDS-AIREN criteria for probable/possible VaD [14]. Patients with the diagnosis of a concomitant neurodegenerative process were excluded from the study. Patients with a history of lobar hemorrhages or space occupying lesions were also excluded.

The study was approved by the Institutional Review Board of Baylor College of Medicine and the MEDVAMC Research and Development Committee.

### *2.2. Baseline assessment*

Dementia diagnoses were made when patients were seen in clinic by two board certified neurologists specializing in neuropsychiatry. They were unaware of the fact that patients with VaD would be part of this future investigation. For this study, the charts of patients diagnosed with VaD were abstracted by a board certified psychiatrist and a senior neurology resident, both unblinded to study design and hypotheses. The abstracted information included a neurologic and medical history (including family history and medication history), findings on neurological examination, laboratory test results, results of brief neuropsychological assessment (e.g., MMSE scores), neuroimaging findings, and multiple demographic variables.

Specifically, the electronic medical records from the first clinical visit were screened for the following past medical illnesses (yes/no): diabetes, hypertension, stroke, dyslipidemia, alcohol abuse/dependence, ischemic heart disease, valvular disease, congestive heart failure, atrial fibrillation and current smoking status (smoker/non-smoker). Electronic medical records from the first clinical visit only were assessed to ensure that the aforementioned medical illnesses were present in the patients prior to the diagnosis of dementia. If any of these medical illnesses developed after the diagnosis of dementia, the association observed between the illness and dementia variables could be spurious. Laboratory results were reviewed to calculate mean HbA1c for all patients with DM, wherever available. HbA1c values recorded at all clinical visits both prior and after the diagnosis of dementia were included. Laboratory HbA1c values were available for only 21 of the total 29 diabetic patients. For the remaining diabetics, average serum glucose values were used to estimate HbA1c using the formula: *Average Glucose* (*mg/dL*) $=$ *28.7xHbA1c-46.7* [15]. Information about vascular dementia (including age of onset of cognitive symptoms, yearly rate of MMSE decline) and patient demographics (age, gender and ethnicity) were recorded for all the patients. Information about 'age of onset'

was abstracted from the electronic record of the first clinical visit of the patient to the Neurology clinic and the values were approximated to the closest year.

Only patients with a baseline MMSE score of 10-24 with at least three follow-up evaluations were selected for further assessment. This range was chosen to be sure that patients had dementia and were within a range where cognitive changes could be accurately ascertained. ‘Yearly rate of MMSE decline’ was determined by calculating the difference between the MMSE score on the first and last visits to the Neurology clinics and dividing the difference by the number of years separating the first and the last clinical visit.

### 2.3. *Assessment of neuropsychiatric symptoms*

The charts of patients with possible/probable VaD were reviewed to identify neuropsychiatric symptoms recorded during their first visit to the Neurology clinic. We specifically examined charts for these symptoms: delusions, hallucinations, anxiety, aggression, euphoria, dis-inhibition, irritability, apathy, aberrant motor activity, and night-time behavioral disturbance. The documented neuropsychiatric symptoms were coded by the nearest symptom (according to the neuropsychiatric inventory, NPI [16]). For example, if the medical record suggested ‘anxious behavior and panic attacks’, the NPI symptom of ‘anxiety’ was coded. Patients were grouped into those *without* neuropsychiatric symptoms and those *with* one or more such symptom. This was done because the small sample size here precluded assessing the relationship between each individual neuropsychiatric symptom and DM. Thus, while we recorded each symptom, for the purposes of analysis, we defined each person as having or not having any neuropsychiatric symptoms.

### 2.4. *Neuroimaging evaluation*

Image assessment was performed by two board certified neurologists reviewing online digital CT/MRI files. The imaging modalities were used to look for evidence of vascular disease as defined by the NINDS-AIREN criteria [14].

### 2.5. *Statistical analysis*

Pearson Chi square tests were used for descriptive analysis and Student’s t tests were used to compare the continuous variables. Multivariate linear regression models were used to test for confounders. Statistical analysis was performed using SPSS version 16.0 (SPSS Inc, Chicago, IL, USA). $P$ values $< 0.05$ were considered significant. Confidence intervals (CIs) were computed based on a 95% confidence level.

For the outcome variables of age of onset and rate of MMSE decline, a minor fraction of the data was missing. Patients for whom the data on more than one outcome variable was missing were excluded from the final analysis.

Table 1
Baseline characteristics of VaD patients with (VaD-DM) and without DM (VaD)

| | VaD-DM ($n = 29$) | VaD ($n = 34$) | p value |
|---|---|---|---|
| | Mean (SD) | | |
| Current Age in years | 77 (7.11) | 79.7 (8.38) | 0.18 |
| Non-Caucasian | 15 (51.7%) | 9 (26.5%) | **0.04** |
| Stroke | 9 (32.1%) | 15 (44.1%) | 0.43 |
| Hypertension | 25 (86.2%) | 26 (76.5%) | 0.36 |
| Dyslipidemia | 21 (72.4%) | 21 (61.8%) | 0.43 |
| Current smokers | 7 (24.1%) | 6 (17.6%) | 0.55 |
| Ischemic heart disease | 13 (44.8%) | 11 (32.4%) | 0.44 |
| Valvular disease | 1 (3.5%) | 0 (0%) | 0.46 |
| Atrial fibrillation | 1 (3.5%) | 3 (8.8%) | 0.62 |
| Congestive heart failure | 7 (24.1%) | 1 (2.9%) | **0.02** |
| Alcoholics | 3 (10.3%) | 6 (17.6%) | 0.49 |

## 3. Results

### 3.1. *Demographics*

Of 471 patients screened for pure VaD, 119 patients fulfilled the NINDS-AIREN criteria. Forty one patients were excluded because of the presence of a concomitant neurodegenerative condition (predominantly AD). Fifteen patients were excluded because the data was missing on more than one outcome variable. Sixty three patients were included in the final analysis. The majority (81%) of patients were males since this is a Veteran population.

The data was dichotomized into two groups based on the presence ($n = 29$) or absence ($n = 34$) of DM. The diabetics and non-diabetics did not differ with regards to age and histories of stroke, hypertension, dyslipidemia, ischemic heart disease, valvular disease, atrial fibrillation, current smoking status or alcohol abuse. A non-Caucasian ethnicity was significantly associated with DM (Table 1).

### 3.2. *Age of onset*

The univariate analysis showed that age of onset for vascular dementia was earlier for diabetics (Table 2).

Table 2
In VaD patients, DM is associated with changes in the age of onset, MMSE decline per year, and neuropsychiatric symptoms

| | VaD-DM (N = 29) | VaD (N = 34) | p value |
|---|---|---|---|
| | Mean (SD) | | |
| Age of onset | 71.9 (6.54) | 77.1 (6.84) | **0.02** |
| MMSE decline/year | 3.60 (1.82) | 2.40 (1.41) | **0.03** |
| One or more neuropsychiatric symptoms | 18 (62.1%) | 3 (12.5%) | < **0.01** |

Table 3
Multivariate association between candidate vascular risk factors and *age of onset* of VaD

| Variables | Regression co-efficient (95% CI) | p value |
|---|---|---|
| Stroke | −4.20 (−8.56,−1.37) | < **0.01** |
| Hypertension | 0.15 (−1.96,7.07) | 0.26 |
| Dyslipidemia | −0.13 (−5.45,1.81) | 0.32 |
| Current Smoker | −0.09 (−5.58,2.67) | 0.48 |
| Ischemic heart disease | −0.03 (−4.14,3.30) | 0.82 |
| Valvular disease | −0.09 (−18.9,8.17) | 0.49 |
| Atrial fibrillation | 0.01 (−7.36,7.88) | 0.95 |
| Congestive heart failure | −0.00 (−5.56,5.50) | 0.99 |
| Alcohol abuse | 0.02 (−4.36,4.96) | 0.89 |
| DM | −4.20 (−9.18,−2.10) | < **0.01** |

The independent effect of individual vascular risk factors on the age of onset of vascular dementia was also studied through multiple linear regression analysis (Table 3). Only a history of DM and stroke were associated with a significantly early onset.

### 3.3. *Yearly decline in MMSE*

Diabetics had a faster rate of decline on MMSE per year on univariate analysis (Table 2). A multiple linear regression analysis of the data also showed that among all the vascular risk factors, only DM was associated with a significantly faster rate of MMSE decline in patients with VaD (Table 4).

### 3.4. *The presence of one or more neuropsychiatric symptoms*

One or more neuropsychiatric symptom was present in 18 of 29 DM patients versus 3 of 34 non-DM patients. The neuropsychiatric symptoms (anxiety, depression, hallucinations, etc.) were grouped because the sample size precluded examining the frequency of individual neuropsychiatric symptoms versus the presence or absence of DM. Grouping all the symptoms demonstrated in the univariate analysis that DM was significantly associated with presence of one or more neuropsychiatric symptoms (Table 2).

Table 4
Multivariate analysis of candidate vascular risk factors and *rate of yearly decline in MMSE*

| Variables | Regression co-efficient (95% CI) | p value |
|---|---|---|
| Stroke | −0.00 (−1.01,0.99) | 0.98 |
| Hypertension | −0.19 (−2.14,0.49) | 0.21 |
| Dyslipidemia | 0.17 (−0.42,1.69) | 0.23 |
| Current Smoker | −0.04 (−1.37,1.02) | 0.77 |
| Ischemic heart disease | 0.04 (−0.93,1.22) | 0.79 |
| Valvular disease | −0.13 (−5.84,2.36) | 0.40 |
| Atrial fibrillation | −0.06 (−2.33,1.53) | 0.68 |
| Congestive heart failure | −0.06 (−1.91,1.32) | 0.72 |
| Alcohol abuse | 0.10 (−0.84,1.86) | 0.45 |
| DM | 0.33 (0.12,2.21) | **0.03** |

### 3.5. *Other analyses*

Spearman's correlations were run between mean HbA1c, age of onset and rate of decline on MMSE. Non-statistically significant negative correlations were observed for HbA1c and age of onset (Spearman's rho $= -0.31$) as well as HbA1c and rate of MMSE decline (Spearman's rho $= -0.08$).

## 4. Discussion

This study suggests that the clinical course of VaD is altered by DM, which was associated with an earlier age of onset of cognitive symptoms, a faster rate of decline in MMSE, and the presence of one or more neuropsychiatric symptoms.

### 4.1. *DM and cognitive decline in VaD*

This study suggests that the presence of DM is associated with an earlier and faster cognitive decline in patients with VaD. The study design here (retrospective) does not allow us to conclude that there is a causal relationship between them. Applying the Bradford Hill's criteria for causation [17] in this scenario, it can be argued that this study shows an evidence for an *association* between DM and altered clinical course of VaD, which is supported by a *biological rationale cohesive* to our current knowledge. However, the retrospective study design precludes any evidence which would have suggested a *temporal relationship*. Future studies will be required to establish a *consistency* of this evidence.

There are pathophysiologic changes in DM that may explain the earlier onset and swifter progression of VaD found in this study. For example, DM has been shown to lead to neuronal injury through a variety of metabolic pathways that may affect endothelial function, protein synthesis, DNA, and mitochondrial function, and may enhance free radical damage and inflammation [18]. Another study, which was autopsy based, demonstrated that elderly patients with dementia and DM had more microvascular infarcts and increased cortical IL-6 concentration [19]. Thus, in addition to producing vascular insults, DM could worsen VaD through a variety of non-vascular mechanisms of neuronal loss or dysfunction.

DM could also worsen VaD by increasing the vascular disease burden at a swifter rate than other risk factors for VaD resulting in earlier onset and faster clinical deterioration. However, this hypothesis remains to be investigated, perhaps by quantifying vascular disease burden in VaD patients with DM versus without DM.

The association between diabetes and worse clinical features of VaD could be interesting from a therapeutic point of view if there is a cause-and-effect relationship between them. A causal relationship, for example, might suggest that better glycemic control could slow the progression of VaD. Interestingly, one finding here seems to weigh against the hypothesis that tighter DM control will mitigate the effects of DM on VaD: there was no significant correlation here between HbA1c and age of onset or the rate of cognitive deterioration in VaD patients. HbA1c is an established indicator of glycemic control. We would caution, however, that this lack of correlation could be due to the small sample size of this study. Alternatively, *recent* HbA1c values were selected, and it is possible that *past* HbA1c values are more relevant. For example, it is possible that glycemic control is more important for preventing the onset of cognitive symptoms than for influencing the disease course once the cognitive deficits have begun.

Another important consideration is that the population studied was predominantly male. A recent study in Italy showed a differential effect of DM on baseline cognitive impairment in men vs. women. Women aged 65–84 years with DM had worse cognition at baseline when compared to non-diabetic women, but did not show worsening at four and eight year follow-up assessments. Men with and without DM did not differ in cognitive performance at baseline, but showed a worsening over time [20]. Interestingly, our male predominant cohort showed both an earlier onset and swifter rate of cognitive decline as assessed by MMSE.

### 4.2. *DM and neuropsychiatric symptoms of dementia*

Neuropsychiatric symptoms are common in VaD patients. They are reported to be present in up to 96.4% of patients [21]. In AD, vascular risk factors, particularly hypertension and stroke, have been associated with an increased risk of neuropsychiatric symptoms [22]. To the best of our knowledge, however, no predictors/correlates for neuropsychiatric symptoms in VaD have been identified. This study suggests that DM may be associated with an increased risk of neuropsychiatric symptoms. This suggests that diabetics with VaD be screened for neuropsychiatric symptoms, such as anxiety or depression, since many can be treated. The retrospective design of this study, however, precludes our recommending such screening at this time. A prospective study will be important to clarify this relationship.

If there are, in fact, more neuropsychiatric symptoms in VaD patients with DM, it suggests that the neuroanatomic dysfunction in DM patients differs from that associated with other etiologies for VaD. Using volumetric analyses, fronto-subcortical changes have been noted in DM that are associated with depression and cognitive changes [23]. It is possible that changes in this area, or like this, led to the increased frequency of neuropsychiatric symptoms noted in our DM patients.

An alternate possibility is that some neuropsychiatric symptoms observed in the DM group are somatopsychic manifestations of DM [24]. The relationships between DM, VaD, and neuropsychiatric symptoms warrant further investigation.

### 4.3. *Diagnostic and therapeutic implications*

The observation that DM is associated with earlier onset and faster cognitive deterioration in VaD is potentially important from a therapeutic and diagnostic point of view. In terms of therapeutics, it raises the question of whether improved glycemic control would improve the outcome for VaD. The results of the ongoing multi-centric trial, Action to Control Cardiovascular Risk in Diabetes Memory (ACCORD-MIND) [18], will be particularly important in this regard. It will assess the impact of strict glycemic, blood pressure and lipid control on the outcomes for validated cognitive measures.

With regard to diagnosis, the earlier onset for VaD in diabetics raises the question of whether they can and should be screened for cognitive changes before VaD develops. There is evidence that cognitive changes are, in fact, present in diabetics before dementia develops. For example, in comparing the cognitive performance of non-demented patients with and without diabetes, diabetic individuals over the age of 80 years perform worse on fronto-subcortical cognitive tasks [25]. Moreover, an MRI-based study of normal elderly suggested that DM is the greatest independent risk factor for deep white matter lesions and those white matter lesions occur more frequently in individuals with DM independent of a history of hypertension [26]. Presumably the gradual accumulation of white matter lesions eventually leads to VaD. Perhaps annual cognitive screening of diabetics, then, would identify persons with early changes in whom VaD might develop subsequently [27].

Support for this suggestion comes from a study by Raji et al. They screened 100 consecutive patient aged $\geqslant 55$ presenting to an eye clinic for routine eye check-ups for cognitive impairment. They found that cognitive deficits were greatest in diabetics [28] suggesting that this population might benefit the most from annual screening examinations. There may also be a role for MRI scans in the screening process. It remains to be demonstrated, however, whether early cognitive or MRI changes progress to VaD, and whether identifying them early is useful for altering the course of VaD.

### 4.4. Conclusions

DM was associated with an earlier onset, faster rate of decline, and the presence of one or more neuropsychiatric symptoms in this study. However, one should be cautious about generalizing the results of this study since it predominantly involved male Veterans, had a retrospective design, and had a modest sample size. Another important consideration is the use of MMSE to gauge cognitive deterioration in this study. Although the tool is widely used, it is known to have a limited sensitivity to cognitive symptoms of dementia subtypes other than AD [29]. Prospective studies will be necessary to corroborate the results of this study and to ascertain whether stricter glycemic control delays the onset and slows the cognitive deterioration in VaD. The association between DM and cognitive changes in other studies and a more malignant course to VaD in this study suggests that cognitive screening for diabetics may be warranted, though future studies will be necessary to determine whether this is valuable. The pathophysiology underlying the enhancement of VaD by DM observed here also remains to be explicated, but appears to have many plausible candidate mechanisms.

## Disclosure

The authors report no conflicts of interest

## Acknowledgements

The authors thank the Lou DeGeorge family for its continued generous support. This material is the result of work supported with resources and the use of facilities at the Michael E. DeBakey Veterans Affairs Medical Center.
